# Presentation and outcomes of chronic kidney disease patients with COVID-19

**DOI:** 10.1590/2175-8239-JBN-2021-0071

**Published:** 2021-11-10

**Authors:** Carolina Gonçalves Branco, Inês Duarte, Joana Gameiro, Cláudia Costa, Filipe Marques, João Oliveira, João Bernardo, José Nuno Fonseca, Carolina Carreiro, Sandra Braz, José António Lopes

**Affiliations:** 1 Centro Hospitalar Universitário Lisboa Norte, Departamento de Medicina, Divisão de Nefrologia e Transplante Renal, Lisboa, Portugal.; 2 Centro Hospitalar Universitário Lisboa Norte, Departamento de Medicina, Divisão de Medicina Interna 2, Lisboa, Portugal.

**Keywords:** COVID-19, Mortality, Renal Insufficiency, Chronic, SARS-CoV-2, COVID-19, Mortalidade, Insuficiência Renal Crônica, SARS-CoV-2

## Abstract

**Introduction:**

COVID-19 is currently a global health issue and an important cause of mortality. Chronic kidney disease (CKD) is one of the risk factors for infection, morbidity and mortality by SARS-CoV-2. In our study, we aimed to evaluate the clinical presentation and outcomes of CKD patients with COVID-19, as well as identify predictors of mortality.

**Methods:**

This was a retrospective study of CKD patients admitted in a tertiary-care Portuguese hospital between March and August of 2020. Variables were submitted to univariate and multivariate analysis to determine factors predictive of in-hospital mortality.

**Results:**

130 CKD patients were analyzed (median age 73.9 years, male 60.0%). Hypertension (81.5%), cardiovascular disease (36.2%), and diabetes (54.6%) were frequent conditions. Cough, dyspnea, fever and respiratory failure were also common. Almost 60% had anemia, 50% hypoalbuminemia, 13.8% hyperlactacidemia and 17% acidemia. Mean serum ferritin was 1531 µg/L, mean CRP 8.3 mg/dL and mean LDH 336.9 U/L. Most patients were treated with lopinavir/ritonavir, hydroxychloroquine or corticosteroids and only 2 with remdesivir. Eighty percent had acute kidney injury and 16.2% required intensive care unit admission. The 34 patients who died were older and more likely to have heart failure. They had higher neutrophils/lymphocytes ratio, ferritin, lactate, and LDH levels. Multivariate analysis identified an association between older age [OR 1.1 (CI 1.01-1.24), p=0.027], higher ferritin [OR 1.0 (CI 1.00-1.00), p=0.009] and higher LDH levels [OR 1.0 (CI 1.00-1.01), p=0.014] and mortality.

**Conclusion:**

In our cohort of CKD patients with COVID-19, older age, higher ferritin, and higher LDH levels were independent risk factors for mortality.

## Resumo

**Introdução:**

COVID-19 é atualmente um problema de saúde global e uma causa importante de mortalidade. Doença renal crônica (DRC) é um dos fatores de risco para infecção, morbilidade e mortalidade por SARS-CoV-2. Neste estudo, objetivamos avaliar a apresentação clínica e os outcomes de doentes com DRC com COVID-19, bem como identificar preditores de mortalidade.

**Métodos:**

Estudo retrospetivo de doentes com DRC internados num hospital terciário português entre Março-Agosto/2020. As variáveis foram submetidas a análise univariada e multivariada para determinar fatores preditivos de mortalidade hospitalar.

**Resultados:**

analisámos 130 pacientes com DRC (média de idades 73,9 anos; 60,0% homens). Hipertensão (81,5%), doença cardiovascular (36,2%) e diabetes (54,6%) foram comorbilidades frequentes. Tosse, dispneia, febre e insuficiência respiratória também foram comuns. Quase 60% apresentavam anemia, 50% hipoalbuminemia e 13,8% hiperlactacidemia, 17% acidemia. A ferritina sérica média foi 1531 µg/L, PCR média 8,3 mg/dL, LDH médio 336,9 U/L. A maioria foi tratada com lopinavir/ritonavir, hidroxicloroquina ou corticosteroides e apenas 2 com remdesivir. Oitenta por cento tiveram lesão renal aguda; 16,2% necessitaram de internamento na unidade de cuidados intensivos. Os 34 pacientes que faleceram eram mais velhos e mais propensos a ter insuficiência cardíaca. Estes apresentaram razão neutrófilos/linfócitos e níveis de ferritina, lactato e LDH mais elevados. A análise multivariada identificou uma associação entre idade avançada [OR 1,1 (IC 1,01-1,24), p=0,027], níveis de ferritina [OR 1,0 (IC 1,00-1,00), p=0,009] e LDH mais elevados [OR 1,0 (IC 1,00-1,01), p=0,014] e mortalidade.

**Conclusão:**

Na nossa coorte de doentes com DRC com COVID-19, a idade avançada e níveis mais elevados de ferritina e LDH foram fatores de risco independentes para mortalidade.

## INTRODUCTION

Coronavirus disease (COVID-19) was first discovered in Wuhan, Hubei Province, China, in December 2019 and declared a global pandemic by March 11th 2020^1^,with 88,828,328 confirmed cases globally by January 11th 2021^2^. Illness severity varies greatly, ranging from asymptomatic and mild clinical course (80%) to severe disease requiring ventilatory support (3-5%)^3^. According to the literature, comorbidities such as cardiovascular disease, hypertension, obesity, diabetes mellitus, cancer and chronic lung disease may contribute to severe disease^4^.

The role of chronic kidney disease (CKD) in COVID-19 was not clear initially. On the one hand, immunosuppression associated to this condition might attenuate the hyperinflammatory state described in COVID-19^5^. On the other hand, immunity dysfunction and high prevalence of comorbidities (including cardiovascular disease and diabetes mellitus) may contribute to a worse clinical course^5^^,^ 6. Recent studies have associated CKD with severe COVID-19, higher risk of hospitalization, and higher mortality^7^^-^ 12.

In summary, given the frequent contact of CKD patients with medical care, which exposes these patients to a higher risk of SARS-CoV-2 infection^13^ and worse disease course, we sought to evaluate the clinical presentation and outcomes of CKD patients with COVID-19, as well as identify predictors of mortality.

## MATERIALS AND METHODS

This study is a retrospective analysis of patients admitted between March and August of 2020 in a unit dedicated for COVID-19 patients in the *Centro Hospitalar Universitário Lisboa Norte* (CHULN) in Lisbon, Portugal named *Unidade de Internamento de Contingência de Infeção Viral Emergente* (UICIVE). The Ethical Committee approved the study, in agreement with institutional guidelines. Informed consent was waived, given the retrospective and non-interventional nature of the study.

### PARTICIPANTS

All adult patients (≥18 years of age) with chronic kidney disease (CKD) and a positive SARS-CoV-2 real time polymerase chain reaction test from nasopharyngeal exudate sample admitted in UICIVE from March 1st to August 31st of 2020 were eligible. For patients who had multiple qualifying hospital admissions, only the first hospitalization was considered. Patients who had less than 2 determinations of serum creatinine (SCr) (a) and patients who were discharged or died less than two days after admission (b) were excluded from the study.

### VARIABLES AND OUTCOMES

Data was obtained from individual electronic clinical records. The following variables were collected: demographic characteristics (age, gender); clinical presentation (cough, fever, dyspnea and respiratory failure); comorbidities [CKD (and previous renal replacement treatment (RRT) need), diabetes mellitus, hypertension, cardiovascular disease (CVD), heart failure, chronic obstructive pulmonary disease (COPD), cirrhosis and/or active malignancy]; current treatment with angiotensin-converting enzyme inhibitors or angiotensin receptor blockers (RAAS inhibitors); disease severity according to Brescia-COVID Respiratory Severity Scale (BCRSS) at admission^14^; laboratory values at admission [serum hemoglobin, hematocrit, neutrophil and lymphocyte count and their ratio (N/L ratio), serum albumin, serum ferritin, SCr (baseline and admission), C-reactive protein (CRP), lactic acid dehydrogenase (LDH), serum sodium, serum chloride, prothrombin time (TP), activated partial thromboplastin time (aPTT), arterial blood gas and pH analysis, and serum lactate]; exposure to nephrotoxins during the first week of admission [non-steroidal anti-inflammatory drugs (NSAIDS), radiocontrast, vancomycin, aminoglycosides]; need for intensive care unit (ICU) admission, mechanical ventilation and vasopressors; acute kidney injury (AKI) development during hospitalization; need for RRT; treatment options used for COVID-19 (hydroxychloroquine, lopinavir/ritonavir, corticosteroids, tocilizumab); length of stay (LOS) and in-hospital mortality.

### DEFINITIONS

COVID-19 diagnosis was established according to the World Health Organization provisional guidelines^15^.

Baseline SCr was defined as a pre-admission value within the previous three months. The estimated glomerular filtration rate (eGFR) of non-dialysis patients was calculated using the Chronic Kidney Disease Epidemiology Collaboration (CKD-EPI) creatinine equation^16^. Presence of CKD was defined as an eGFR lower than 60 mL/min/1.73m^2^. AKI was defined and stratified using SCr criteria of Kidney Disease Improving Global Outcomes (KDIGO) classification^17^.

Diabetes *mellitus* was defined in accordance with the American Diabetes Association Guidelines^18^. Arterial hypertension was diagnosed according to the European Society of Cardiology and European Society of Hypertension Guidelines^19^. COPD included emphysema and chronic bronchitis. If a history of cerebrovascular disease, chronic heart failure of any cause, ischemic heart disease and/or peripheral arterial disease was documented, CVD was considered. Acidemia was defined as blood gas pH below 7.35.

### STATISTICAL METHODS

Categorical variables were described as total number and percentage of each category, while continuous variables were described as mean ± standard deviation. Continuous variables were compared using Student's t-test and categorical variables were compared using Chi-square test.

All variables were submitted to univariate analysis to find statistically significant factors that could be predictive of in-hospital mortality. Subsequently, variables with a significant association underwent multivariate analysis using the Cox-logistic regression method. Data are reported as odds ratios (OR) with 95% confidence intervals (CI). Statistical significance was established as a P-value lower than 0.05. The statistical software SPSS for Windows (version 21.0) was used for data analysis.

## RESULTS

A total of 130 CKD patients were admitted to UICIVE with a diagnosis of COVID-19.

### BASELINE CHARACTERISTICS

Baseline characteristics of this cohort are described in Table 1. Mean age was 73.9 ±12.2 years and the majority of patients were male (60.0%). There was a large prevalence of hypertensive (81.5%), CVD (54.6%), heart failure (51.5%) and diabetic (36.2%) patients. Twenty-four patients (18.5%) were on hemodialysis and 3 patients (2.3%) had a kidney transplant. Forty five percent of the patients were taking RAAS inhibitors. Mean baseline serum creatinine (SCr) was 1.7±0.9 mg/dL and mean baseline eGFR was 42.5±15.6 mL/min/1.73m^2^.

**Table 1:** Patients’ baseline characteristics and in-hospital mortality

Characteristic	Total (n=130)	Mortality (n=34)	Survival (n=96)	p-value
Age (year)	73.9 ± 12.2	83.1 ± 10.3	70.6 ± 18.1	0.000
Gender (Male) – n (%)	78 (60.0)	23 (67.6)	55 (57.3)	0.290
Comorbidities – n (%)				
Hypertension	106 (81.5)	27 (79.4)	79 (82.3)	0.710
Diabetes	47 (36.2)	13 (38.2)	34 (35.4)	0.769
CVD	71 (54.6)	20 (58.8)	51 (53.1)	0.566
Heart failure	67 (51.5)	24 (70.6)	43 (44.8)	0.010
COPD	19 (14.6)	6 (17.6)	13 (13.5)	0.578
Cirrhosis	8 (6.2)	1 (2.9)	7 (7.3)	0.680
Neoplasia	21 (16.2)	9 (26.5)	12 (12.5)	0.057
CKD on RRT – n (%)	27 (20.8)	5 (14.7)	22 (22.9)	0.443
Hemodialysis	24 (18.5)	5 (14.7)	19 (52.8)	
Kidney transplant	3 (2.3)	0 (0.0)	3 (3.1)	
RAAS inhibitors – n (%)	58 (44.6)	12 (35.3)	46 (47.9)	0.305
Nephrotoxic agent – n (%)	16 (12.3)	3 (8.8)	13 (15.5)	0.760
Baseline SCr (mg/dL)	1.7 ± 0.9	1.7 ± 1.0	1.7 ± 0.9	0.831
Baseline eGFR (mL/min/1.73m2)	42.5 ± 15.6	41.1 ± 16.9	43.0 ± 15.2	0.561
Brescia Score >2 – n (%)	17 (13.1)	10 (29.4)	7 (7.3)	0.000
Clinical presentation – n (%)				
Cough	60 (46.2)	15 (44.1)	38 (39.6)	0.644
Fever	49 (37.7)	12 (35.3)	37 (38.5)	0.737
Dyspnea	60 (46.2)	23 (67.6)	37 (38.5)	0.003
Respiratory failure	57 (43.8)	21 (61.8)	36 (37.5)	0.016
Laboratory				
Admission SCr (mg/dL)	3.3 ± 3.5	3.2 ± 3.9	3.4 ± 3.3	0.758
Hemoglobin (g/dL)	11.7 ± 2.3	11.2 ± 2.6	11.9 ± 2.2	0.136
Anemia – n (%)	74 (56.9)	24 (70.6)	50 (52.1)	0.061
Hematocrit	35.9 ± 6.7	34.3 ± 7.7	36.6 ± 6.2	0.098
NL ratio	7.2 ± 6.1	9.4 ± 5.7	6.4 ± 6.0	0.012
Serum albumin (g/dL)	3.3 ± 0.5	4.0 ± 0.0	3.3 ± 0.5	0.170
Hypoalbuminemia – n (%)	66 (50.8)	18 (52.9)	48 (50.0)	0.973
Serum ferritin (ug/dL)	1531.9 ± 2580.5	3183.9 ± 4248.3	958.7 ± 1303.6	0.038
CRP (mg/dL)	8.3 ± 8.9	9.8 ± 9.5	7.8 ± 8.7	0.261
Acidemia – n (%)	22 (16.9)	7 (20.6)	15 (15.6)	0.528
Lactate level (mg/dL)	13.8 ± 8.6	16.5 ± 10.2	12.8 ± 7.8	0.038
Hyperlactacidemia – n (%)	18 (13.8)	8 (23.5)	10 (10.4)	0.106
LDH level (mg/dL)	336.9 ± 225.1	453.2 ± 362.2	295.7 ± 129.2	0.000
Serum sodium (mmol/L)	137.7 ± 7.7	138.2 ± 6.4	137.5 ± 8.1	0.624
Serum chloride (mmol/L)	106.6 ± 7.9	107.0 ± 7.4	106.4 ± 8.2	0.718
TP	15.6 ± 9.8	16.1 ± 5.9	15.4 ± 10.7	0.764
aPTT	30.6 ± 6.4	30.8 ± 6.3	30.6 ± 6.5	0.911
ICU admission – n (%)	21 (16.2)	8 (23.5)	13 (13.5)	0.174
Mechanical ventilation – n (%)	11 (8.5)	4 (11.8)	7 (7.3)	0.729
Vasopressor use – n (%)	5 (3.8)	2 (5.9)	3 (3.1)	0.626
COVID-19 treatment				
Hydroxychloroquine – n (%)	27 (20.8)	12 (35.3)	15 (15.6)	0.012
Lopinavir/ritonavir – n (%)	36 (27.7)	10 (29.4)	26 (27.1)	0.794
Corticosteroids – n (%)	23 (17.7)	5 (14.7)	18 (18.8)	0.641
Remdesivir – n (%)	2 (1.5)	0 (0.0)	2 (2.1)	0.552
AKI – n (%)	105 (80.8)	29 (85.3)	76 (79.2)	0.436
RRT – n (%)	28 (21.5)	8 (23.5)	20 (20.8)	0.954
LOS in hospital (days)	35.0 ± 45.9	31.1 ± 49.5	36.4 ± 44.9	0.567

### CLINICAL PRESENTATION AND LABORATORIAL FINDINGS AT ADMISSION

The main clinical presentation was cough and dyspnea in 46.2% of patients each, respiratory failure in 43.8%, and fever in 37.7% of patients. Thirteen percent of patients had a Brescia score greater than 2.

At admission, mean SCr was 3.3±3.52 mg/dL, mean hemoglobin was 11.7±2.3 g/dL with almost 60% of patients being anemic, mean N/L ratio was 7.2±6.1, mean serum albumin was 3.3±0.5g/dL with more than half of the patients with hypoalbuminemia. Mean serum ferritin was 1531.9±2580.5 µg/L and mean CRP was 8.3±8.9 mg/dL. Mean lactate level was 13.8±8.6 mg/dL with hyperlactatemia in 13.8% (n=18) of patients and acidemia in 16.9% (n=22). Mean LDH level was 336.9±225 U/L, mean serum sodium 137.7±7.7 mmol/L, and mean chloride 106.6±7.9 mmol/L. Mean prothrombin (TP) time was 15.6±9.8 s and mean activated partial thromboplastin time (aPTT) 30.6±6.4 s.

Concerning treatment, a vast majority of patients were taking lopinavir/ritonavir (27.7%), hydroxychloroquine (20.8%), and corticosteroids (17.7%). Only 2 patients were treated with remdesivir.

One hundred and five patients (80.8%) developed AKI during hospital stay and 28 required dialysis, although only twelve percent of patients were exposed to nephrotoxins during hospitalization.

Sixteen percent of patients (n=21) required admission in ICU, 8.5% of patients (n=11) mechanical ventilation, and 3.8% (n=5) vasopressor use.

### IN-HOSPITAL MORTALITY

LOS was 35.0±45.9 days and 26.2% of patients died in hospital (n=34). Patient characteristics according to in-hospital mortality are described in Table 1.

Patients who died were significantly older [83.1±10.3 vs 70.6±18.1, p=0.000; unadjusted OR (uOR) 1.1 (CI 1.03-1.10), p=0.001] and were more likely to have pre-existing heart failure [70.6% vs 44.8%, p=0.010; uOR 2.9 (CI 1.28-6.85), p=0.011] than those who survived.

At admission, these patients also presented with higher N/L ratios [9.4±5.7 vs 6.4±6.0, p=0.012; uOR 1.1 (CI 1.01-1.15). p=0.017], higher ferritin levels [3183.9±4248.3 vs 958.7±1303.6, p=0.038; uOR 1.0 (CI 1.00-1.01), p=0.021], higher lactate levels [16.5±10.2 vs 12.8±7.8, p=0.038; uOR 1.1 (CI 1.00-1.09), p=0.048] and higher LDH levels [453.2±362.2 vs 295.7±129.2, p=0.000; uOR 1.0 (CI 1.00-1.01), p=0.005].

On multivariate analysis, age [adjusted OR (aOR) 1.1 (CI 1.01-1.24), p=0.027], ferritin level at admission [aOR 1.0 (CI 1.00-1.00), p=0.009], and LDH level [aOR 1.0 (CI 1.00-1.01), p=0.014] were independent predictors of in-hospital mortality in CKD patients (Table 2).

**Table 2:** Univariate and multivariate analysis of factors predictive of mortality in chronic kidney disease COVID-19 patients

Characteristic	Mortality			
	Unadjusted OR (95% CI)	P-value	Adjusted OR (95% CI)	P-value
Age (year)	1.1 (1.03 – 1.10)	0.001	1.1 (1.01 – 1.24)	0.027
Gender (Male) – n (%)	1.6 (0.68 – 3.56)	0.291		
Comorbidities – n (%)				
Hypertension	0.8 (0.31 – 2.22)	0.710		
Diabetes	1.2 (0.50 – 2.53)	0.769		
CVD	1.3 (0.57 – 2.78)	0.567		
Heart failure	2.9 (1.28 – 6.85)	0.011	2.0 (0.36 – 10.93)	0.426
COPD	1.4 (0.48 – 3.94)	0.561		
Cirrhosis	0.4 (0.05 – 3.25)	0.381		
Neoplasia	2.5 (0.95 – 6.67)	0.063		
RAAS inhibitors – n (%)	0.7 (0.29 – 1.48)	0.307		
Nephrotoxic agent – n (%)	0.6 (0.17 – 2.39)	0.506		
Baseline SCr (mg/dL)	1.0 (0.69 – 1.59)	0.829		
Laboratory				
Admission SCr (mg/dL)	0.9 (0.87 – 1.11)	0.756		
Hemoglobin (g/dL)	0.9 (0.74 – 1.04)	0.137		
Hematocrit (%)	0.9 (0.89 – 1.01)	0.099		
NL ratio	1.1 (1.01 – 1.15)	0.017	1.1 (0.95 – 1.24)	0.213
Serum ferritin (µ g/dL)	1.0 (1.00 – 1.01)	0.021	1.0 (1.00 – 1.00)	0.009
CRP (mg/dL)	1.0 (0.98 – 1.07)	0.262		
Lactate level (mg/dL)	1.1 (1.00 – 1.09)	0.048	1.0 (0.93 – 1.11)	0.710
LDH level (mg/dL)	1.0 (1.00 – 1.01)	0.005	1.0 (1.00 – 1.01)	0.014
ICU admission – n (%)	1.9 (0.73 – 5.26)	0.179		
Mechanical ventilation – n (%)	1.4 (0.38 – 5.19)	0.607		
Vasopressor use – n (%)	1.7 (0.27 – 10.57)	0.581		
AKI – n (%)	1.5 (0.52 – 4.45)	0.438		
RRT – n (%)	1.0 (0.39 – 2.69)	0.954		

## DISCUSSION

After more than a year since the first COVID-19 reported case, our knowledge of the disease characteristics in the general population have grown substantially. However, the clinical presentation of SARS-CoV-2 infection in CKD patients is not as described in the literature, although there seems to be an association of this comorbidity with mortality and severe presentation^11^^,^ 12.

In our study, cough, dyspnea, and fever were frequent symptoms, which is in line with what has been previously reported in CKD patients (cough in 35-69%, dyspnea in 6.25-57%, and fever in 43-71%)^3^^,^ 13 ^,^ 20 ^-^ 24, with the exception of the ERACODA series that documented considerably lower incidences (cough in 9.55%, dyspnea in 6.65%, and fever in 11%). This could be explained by the inclusion of CKD patients followed and treated as outpatients and, therefore, more likely to be asymptomatic. Our findings are also in line with what is seen in the general population^25^^-^ 28.

Regarding laboratorial findings at hospital admission, we found that more than half of our patients had anemia, although with a higher mean hemoglobin than reported in previous cohorts^3^^,^ 21 ^,^ 24 ^,^ 29. As these studies included mainly hemodialysis patients, the insufficient endogenous production of erythropoetin and chronic inflammatory status, as well as the exposure to heparin during treatment may explain the discrepancy^21^. Hypoalbuminemia was common in our cohort, but with lower mean values than previously described (3.4-3.7 g/dL)^3^^,^ 9 ^,^ 22 ^,^ 24. This might indicate a worse nutritional status of our cohort. As expected, inflammation markers (ferritin and CRP) and LDH were elevated^3^^,^ 4 ^,^ 9 ^,^ 20 ^,^ 22 ^-^ 24 ^,^ 26 ^,^ 29.

During the first half of our investigation period, national guidelines recommended treating COVID-19 patients with pneumonia and/or respiratory failure with hydroxychloroquine and/or lopinavir/ritonavir (or remdesivir in the ICU setting)^30^. This, in conjunction with the potential risks associated with the administration of remdesivir in patients with a eGFR lower than 30 mL/min, explains the frequencies of treatment described in our cohort^5^.

Several studies have recorded a broad range of AKI incidence in COVID-19 in the general population (0.5-46%)^31^. CKD is a risk factor for AKI, which explains why it was frequent in our patients^31^.The high percentage of lopinavir/ritonavir use and severe presentations could have also contributed to this fact^32^. As this cohort included CKD patients already under RRT, the effect of the need for *de novo* RRT in mortality might have been underestimated.

There is a wide range of ICU admission (12-39.4%)^4^^,^ 9 ^,^ 23 and mechanical ventilation (4-31.5%)^4^^,^ 9 ^,^ 13 ^,^ 22 ^-^ 24 ^,^ 29 rates in the literature depending on the series and the relative percentage of CKD patients under RRT. In our cohort, 16% of CKD patients where admitted in ICU, 8.5% were mechanically ventilated, and 3.8% needed vasopressors. These relatively low percentages could be explained by the older age of our patients and the resulting higher burden of comorbidities and clinical frailty as well as by the inclusion of CKD patients who were already under RRT at admission, possibly underestimating the effect of mechanical ventilation on mortality.

Twenty six percent of our patients died, which is similar to what has been documented in inpatients (11.1-42.0%)^3^^,^ 6 ^,^ 9 ^,^ 13 ^,^ 22 ^-^ 24 ^,^ 29 ^,^ 33 ^-^ 35. Older age, male sex, undocumented status, obesity, higher comorbidity index, frailty, longer dialysis vintage, symptoms and signs such as dyspnea, cough, higher body temperature, higher respiratory/pulse rate and lower oxygen saturation, severe presentation, need for mechanical ventilation, laboratorial alterations such as anemia, higher levels of white blood-cells count, lymphopenia, liver enzymes, LDH, CRP, ferritin and interleucin-6, abnormal kidney function and lower albumin, and prednisone use have been associated to mortality in the CKD population^3^^-^ 5 ^,^ 13 ^,^ 22 ^,^ 24 ^,^ 29 ^,^ 33 ^,^ 35 ^,^ 36.

We found that the deceased were significantly older, had higher NL ratios and ferritin, lactate and LDH levels. There was also an association between death and heart failure. However, after a multivariate analysis, only older age, higher ferritin, and higher LDH levels were independent risk factors for mortality. Older age is a well-documented risk factor for infection, morbidity, and mortality by SARS-CoV-2, as it negatively affects lung function and immunity response^8^^,^ 10. SARS-CoV-2 infection induces a pro-inflammatory state, which can lead to cytokine storm response and subsequently to secondary tissue damage and a poorer prognosis^37^^,^ 38. As ferritin acts as an acute phase protein and LDH as a marker for tissue damage, we hypothesize there may be a connection between their elevation and hyperinflammatory response in COVID-19.

Chawki S. et al. also found reduced mortality in CKD patients treated with RAAS inhibitors^33^. This is a controversial subject in the literature, with description of potential upregulation of angiotensin-converting enzyme 2 receptor, as well as its blockage^31^^,^ 33. Most studies in the general population have failed to show an association between RAAS and mortality, and professional societies continue recommending its use^31^^,^ 33. In our cohort no association was found.

We must take into account several limitations of our study. First, this was a single-center, retrospective study, which limits generalization of results. The small size of our cohort and the lack of some laboratorial results may have compromised, at least in part, our conclusions. Additionally, causes of CKD were not assessed. Regardless of these potential biases, there are some strengths worth noting. The most important is the study population, which included all stages of CKD, not only end-stage kidney disease as in most published series. Furthermore, to the best of our knowledge, this is the largest study of COVID-19 in CKD patients in Portugal^39^^,^ 40.

To conclude, in this cohort of CKD patients with COVID-19, older age and higher ferritin and LDH levels at admission were independent risk factors for mortality, suggesting their potential use as predictors of poorer prognosis.
